# The entire chloroplast genome sequence of *Asparagus cochinchinensis* and genetic comparison to *Asparagus* species

**DOI:** 10.1515/biol-2022-0098

**Published:** 2022-08-10

**Authors:** Wentao Sheng

**Affiliations:** Department of Biological Technology, Nanchang Normal University, Nanchang 330032, Jiangxi, China

**Keywords:** *Asparagus cochinchinensis*, chloroplast genome, *Asparagus*, phylogenetic analysis, molecular identification

## Abstract

*Asparagus cochinchinensis* is a traditional Chinese medicinal plant. The chloroplast (cp) genome study on *A. cochinchinensis* is poorly understood. In this research, we collected the data from the cp genome assembly and gene annotation of *A. cochinchinensis*, followed by further comparative analysis with six species in the genus *Asparagus*. The cp genome of *A. cochinchinensis* showed a circular quadripartite structure in the size of 157,095 bp, comprising a large single-copy (LSC), a small single-copy (SSC), and two inverted repeat (IR) regions. A total of 137 genes were annotated, consisting of 86 protein-coding genes, 8 ribosomal RNAs, 38 transfer RNAs, and 5 pseudo-genes. Forty scattered repetitive sequences and 247 simple sequence repeats loci were marked out. In addition, A/T-ending codons were shown to have a basis in the codon analysis. A cp genome comparative analysis revealed that a similar gene composition was detected in the IR and LSC/SSC regions with *Asparagus* species. Based on the complete cp genome sequence in Asparagaceae, the result showed that *A. cochinchinensis* was closely related to *A. racemosus* by phylogenetic analysis. Therefore, our study providing *A. cochinchinensis* genomic resources could effectively contribute to the phylogenetic analysis and molecular identification of the genus *Asparagus*.

## 1 Introduction

Chloroplast (cp) is a photosynthetic organelle that is strictly determined by heredity in plant cells and has its own complete genome. The cp genome is usually maternal-inherited in angiosperm plants, mainly used to encode some electron chain transfer functional proteins and ribosomal structure proteins associated with photosynthesis in plant organs [1]. The cp genome of terrestrial angiosperm plants is usually 120–160 kb in sequence length. A typical quadripartite structure was exhibited in its genome, constituting a large single-copy (LSC) region, three inverted repeat regions (IRs, IRa, and IRb), and a small single-copy (SSC) region [2]. The cp genome structure of plant species is conservative to some extent, but the sequence composition of the cp genome is different between species, especially in non-coding areas [3]. The cp genome of maternal inheritance is not easy to recombine, and it is easier to analyze than the nuclear genome in genomic characteristics, which is more helpful in solving the problems of taxonomic and molecular evolution issues with these variable sequence fragments [4].

*Asparagus cochinchinensis* is an important resource plant of traditional Chinese medicine, belonging to the genus *Asparagus* of Asparagaceae. [5]. It has thousands of years of medicinal history in China and was recorded in the Compendium of Materia Medica, with the prominent effects treating fever, cough and vomiting, sore throat, constipation, and other diseases [6,7]. Up to now, research on *A. cochinchinensis* is mainly focused on chemical constituents, pharmacological effects, and clinical applications [8]. In spite of its medicinal importance, there was limited genomic sequence information released for *A. cochinchinensis*. The cp genome study on *A. cochinchinensis* is very few. Furthermore, no comprehensive, systematic, and comparative studies were reported in its genome structure and gene sequence constitution. Within the genus *Asparagus*, the complete cp genomes of five species (*A. filicinus*, *A. officinalis*, *A. racemosus*, *A. schoberioides*, and *A. setaceus*) have been registered in GenBank, thus providing potential genetic information for cp genome comparison and phylogenetic evolution analysis in the genus.

In the present research, the entire cp genome of *A. cochinchinensis* was de novo sequenced and assembled with the Illumina sequencing platform to illustrate its structure characteristics. In addition to genome annotation and genetic comparison, sequence diversification studies of *Asparagus* species based on the new assembly with reported cp genomes are also identified to deepen our understanding of *A. cochinchinensis* cp genome and provide insights into its molecular evolutionary relationship.

## 2 Methods

### 2.1 Plant material

Plant seeds of *A. cochinchinensis* were kindly provided by Guangyu Chen (Jiangxi, China) and germinated at room temperature at Nanchang Normal University. Total genomic DNA of its tender phyllodes was extracted using the Dneasy Plant Mini Kit (Tiangen Biotech, Beijing, China) according to the manufacturer’s instructions. After the DNA concentration and purity test were qualified, the sequencing library was constructed by random breaking into 350 bp by mechanical interrupt method. The Illumina HiSeq PE150 platform was used for double-end sequencing of the DNA library at Genepioneer Biotechnologies (Nanjing, China). The sequencing depth was ten times.

### 2.2 Cp genome assembly and annotation

The quality of the original sequencing data was assessed using FastQC v0.11.7 software [9]. After quality assessment, all original readings were compared with the reported cp genome in the genus, and the cp reads of *A. cochinchinensis* were extracted. Based on the comparison results, the sequence with the best coverage was selected as the optimal reference sequence. SOAPdenovo2 was used to assemble all relevant reads into overlapping groups, and the assembly results were optimized according to the paired-end and overlap relationships of reads [10]. Finally, NOVO-Plasty was used to fill the holes in the assembly results [11].

We used two methods to annotate the cp genome. First, prodigal v2.6.3 (https://www.github.com/hyattpd/Prodigal) was used to annotate coding DNA sequences (CDs), hmmer v3.1b2 (http://www.hmmer. org/) was used to predict rRNA, and Aragorn v1.2.38 (http://130.235.244.92/ARAGORN/) was used to predict tRNA. Second, according to the related species published in NCBI, the gene sequence was extracted, and then Blast v2.6 (https://blast.ncbi.nlm.nih.gov/Blast.cgi) was used to compare the assembled sequence to obtain the second annotation result. Then, the two annotation results were manually checked for genes with differences, and the wrong and redundant annotations were removed to determine the boundaries of multiple exons to obtain the final annotation result. In addition, Sequin software in NCBI was used to complete the submission of *A. cochinchinensis* cp genome. The OGDRAW online tool was made to draw the circular map of cp genome in *A. cochinchinensis* [12].

### 2.3 Characterization analysis of repeat sequences

Simple sequence repeats (SSRs) markers are a class of tandem repeats consisting of several nucleotides (generally 1–6) as repeat units. SSRs were identified with the MISA software based on the following minimum number: 10, 5, 4, 4, 4, 3 for 1–6 repeat unit nucleotides, respectively [13]. Sporadic repeats are another kind of repeats different from tandem repeats. Combined with Perl script, we used Vmatch v2.3.0 (http://www.vmatch.de/download.html) to discriminate repetitive sequences. The parameters were set as follows: minimum length = 30 bp, Hamming distance = 3, sequence identity was >90%, and there were four identification forms: forward, reverse, palindromic, and complement repeats.

### 2.4 Codon usage bias (CUB)

The relative synonymous codon usage frequency (RSCU) was calculated for *A. cochinchinensis* cp genome in the present study. Eighty-six CDs were obtained from the cp genome in total. In addition to the repetitive gene sequences and sequences less than 300 bp in length, the remaining 53 CDs were used for CUB analysis with CodonW1.4.2 (http://downloads.fyxm.net/CodonW-76666.html).

### 2.5 Cp comparative genome analysis

Based on the cp genome annotation available in NCBI (https://www.ncbi.nlm.nih.gov/), we compared the joining area of LSC, SSC, IRa, and IRb boundaries among the sequencing *Asparagus* species. The program MAUVE v1.1.1 was used to analyze the structural variation in the whole genome and to detect the gene order rearrangements and IR expansion/contraction [14]. In order to calculate the synonymous (Ks) and non-synonymous (Ka) substitution rates of the cp genome, the protein-coding genes of *A. cochinchinensis* were compared with five reported *Asparagus* species. We aligned the corresponding protein-coding genes using MAFFT [15]. And the Ks, Ka, and Ka/Ks values were computed with Ka/Ks calculator software (https://sourceforge.net/projects/360 kakscalculator2/).

### 2.6 Molecular phylogenetic analysis

Based on the APG classification system, Asparagaceae can be divided into Asparagoideae, Aphyllanthoideae, Agavoideae, Hyacinthaceae, Nolinoideae, Lomandroideae, and Brodiaeoideae [16]. For the phylogenetic maximum-likelihood (ML) analysis, we downloaded the cp genomes of 19 plant species belonging to Asparagaceae, and *Allium cyaneum* (NC_058219.1) of Amaryllidaceae was selected as the outgroup species from GenBank to access the evolutionary relationship of *A. cochinchinensis.* The complete cp DNA sequences were aligned with MAFFT v7 under default parameters [15], and the resulting alignments were trimmed with Gblocks (get_gblocks_trimmed_alignment_from_untrimmed.py, settings: b1 = 0.5, b2 = 0.5, b3 = 12, b4 = 7) [17]. After trimming, the ML method was performed for the genome-wide phylogenetic analysis using PhyML 3.0 [18]. The model GTR + I + G were used for ML analyses with 1,000 bootstrap replicates to calculate the bootstrap values of the topology.

## 3 Results

### 3.1 Cp genome characteristics in *A. cochinchinensis*

**Figure 1:**
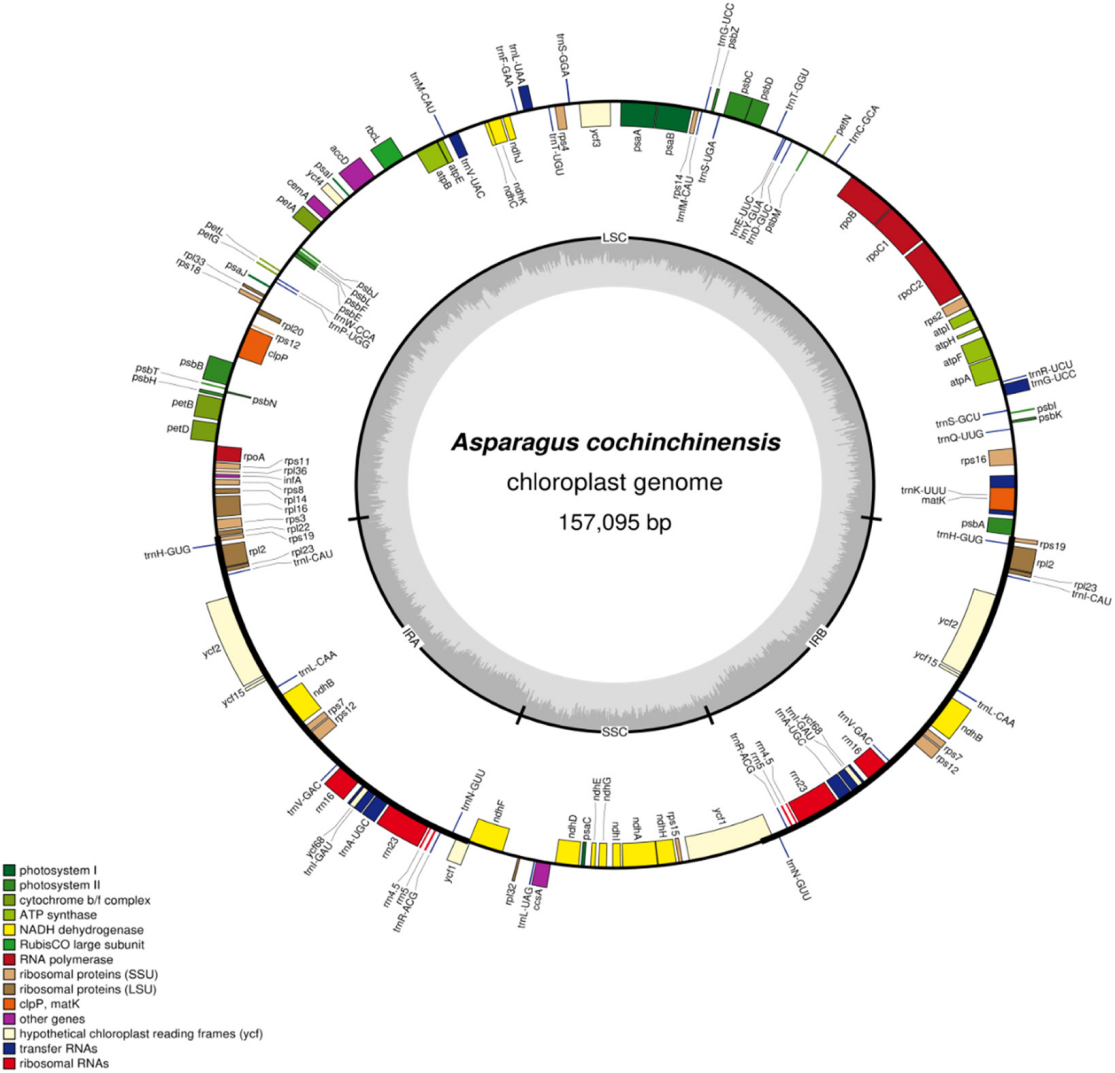
The cp genome map of *A. cochinchinensis*. Genes are located outside of the circle and inside of the circle, which are transcribed clockwise and counterclockwise, respectively. The outermost ring represented the genome sequence; the coding gene is expressed in the green frame; tRNA is expressed in the purple frame; rRNA is expressed in the orange frame; the green ring inside shows the coverage depth, and the depth of reverse repeat area is generally twice as deep as other areas; the darker gray are corresponding to GC content in the inner ring. The areas are marked in the circle, including LSC, SSC, IRa, and IRb regions.

The whole genome sequence was obtained based on high-throughput sequencing. After removing low-quality reads, approximately 10 Gb sequence data of clean data were obtained. The guanine-cytosine (GC) content of the whole genome sequence was 36.58%, the Q20 value of effective data accounted for was 97.56%, and the Q30 value of effective data accounted for was 93.17%. According to the comparison coverage, it was found that *A. officinalis* (NC_034777.1) was the best reference sequence, with a coverage of 94.77%. After the assembled cp genome was tested and qualified, the complete genome was registered in GenBank with the sequence number MW447164.1. And the circular cp genome of *A. cochinchinensis* exhibited a conserved quadripartite structure, including the LSC region (85,306 bp), SSC region (18,677 bp), IRa, and IRb regions (53,112 bp) (Figure 1). The GC content observed in the cp genome, SSC, LSC, and IR regions were 37.48, 31.30, 35.45, and 42.93%, respectively.

In the *A. cochinchinensis* cp genome, 137 unique genes with the same combination pattern were annotated, comprising 86 protein-coding genes, 38 tRNAs, 8 rRNAs, and 5 pseudo-genes. Forty-five genes were related to photosynthesis in the cp genome, including the encoding subunits of photosystem I and II, subunits of rubisco, subunits of NADH dehydrogenase, subunits of ATP synthase, cytochrome b/f complex, and c-type cytochrome synthetase. Seventy-three genes were associated with self-replication functions, which were composed of 12 large ribosomal subunit protein genes, 15 small ribosomal subunit protein genes, 4 RNA polymerase subunit protein genes, 4 rRNA genes, and 38 tRNA genes. Furthermore, some other genes were also annotated containing *matK*, *clpP*, *cemA*, *accD*, *ccsA*, *infA*, and genes of unknown function (*ycf1*, *ycf15*, *ycf68*, *ycf2*, *ycf3*, and *ycf4*) (Table 1).

**Table 1:** The list of gene categories annotated in the cp genome of *A. cochinchinensis*

Category	Gene group	Gene name
Photosynthesis	Subunits of photosystem I	*psaA, psaB, psaC, psaI,* and *psaJ*
	Subunits of photosystem II	*psbA, psbB, psbC, psbD, psbE, psbF, psbH, psbI, psbJ, psbK, psbL, psbM, psbN, psbT,* and *psbZ*
	Subunits of NADH dehydrogenase	ndhA*, ndhB*(2), ndhC, ndhD, ndhE, ndhF, ndhG, ndhH, ndhI, ndhJ, and *ndhK*
	Subunits of cytochrome b/f complex	petA, petB*, petD*, petG, petL, and *petN*
	Subunits of ATP synthase	atpA, atpB, atpE, atpF*, atpH, and *atpI*
	Large subunit of rubisco	*rbcL*
	Subunits photochlorophyllide reductase	*–*
Self-replication	Proteins of large ribosomal subunit	rpl14, rpl16*, rpl2*(2), rpl20, rpl22, rpl23(2), rpl32, rpl33, and *rpl36*
	Proteins of small ribosomal subunit	rps11, rps12**(2), rps14, rps15, rps16*, rps18, rps19(2), rps2, rps3, rps4, rps7(2), and *rps8*
	Subunits of RNA polymerase	rpoA, rpoB, rpoC1*, and *rpoC2*
	Ribosomal RNAs	*rrn16(2), rrn23(2), rrn4.5(2),* and *rrn5(2)*
	Transfer RNAs	trnA-UGC*(2), trnC-GCA, trnD-GUC, trnE-UUC, trnF-GAA, trnG-UCC, trnG-UCC*, trnH-GUG(2), trnI-CAU(2), trnI-GAU*(2), trnK-UUU*, trnL-CAA(2), trnL-UAA*, trnL-UAG, trnM-CAU, trnN-GUU(2), trnP-UGG, trnQ-UUG, trnR-ACG(2), trnR-UCU, trnS-GCU, trnS-GGA, trnS-UGA, trnT-GGU, trnT-UGU, trnV-GAC(2), trnV-UAC*, trnW-CCA, trnY-GUA, and *trnfM-CAU*
Other genes	Maturase	*matK*
	Protease	clpP**
	Envelope membrane protein	*cemA*
	Acetyl-CoA carboxylase	*accD*
	c-type cytochrome synthesis gene	*ccsA*
	Translation initiation factor	*infA*
	other	*–*
Genes of unknown function	Conserved hypothetical chloroplast ORF	#ycf1, #ycf15(2), #ycf68(2), ycf1, ycf2(2), ycf3**, and *ycf4*

Gene*: gene included one intron; Gene**: gene included two introns; #Gene: pseudogene; Gene (2): gene associated with copy number >1, and the copy number is marked in brackets.

Six protein-coding genes, nine tRNAs, and four rRNAs were shown to contain two copies in this genome. Meanwhile, we identified that 21 genes (11 protein-coding genes and 10 tRNAs) had one intron and 3 genes (*ycf3*, *rps12*, and *clpP*) owned two introns (Table 1). Furthermore, the *ycf1, ycf15,* and *ycf68* genes were identified as pseudogenes, of which *ycf15* and *ycf68* included two copies. *Rps12* was also found to be a trans-spliced gene owning a 5′-end located in the LSC region and a 3′-end duplicated in the IR region. In particular, the largest intron (2,579 bp) was found to be within the *trnK-UUU* gene, and the smallest intron (527 bp) was positioned in the *trnL-UAA* gene. It was revealed that introns were usually larger than exons in sequence size (Table 2). Meanwhile, the SSC part possessed 12 protein-coding genes and 1 tRNA gene, while the LSC part contained 58 protein-coding genes and 21 tRNA genes. In addition, 16 tRNA, 8 rRNA, and 22 protein-coding genes were positioned in the IRa and IRb parts. Moreover, 24 genes with introns were detected, among which 5 genes (*rps12*, *rpl2*, *ndhB*, *trnI-GAU*, and *trnA-UGC*) were located in the IRs region (Figure 1, Table 2).

**Table 2:** The exon and intron length in *A. cochinchinensis* cp gene

Gene	Position	Exon I (bp)	Intron I (bp)	Exon II (bp)	Intron II (bp)	Exon III (bp)
*trnK-UUU*	LSC	37	2,579	35		
*rps16*	LSC	40	858	209		
*trnG-UCC*	LSC	24	676	48		
*atpF*	LSC	144	854	411		
*rpoC1*	LSC	436	751	1,625		
*ycf3*	LSC	126	749	229	721	152
*trnL-UAA*	LSC	35	527	50		
*trnV-UAC*	LSC	39	596	35		
*rps12*	IRa	126	—	229	544	26
*clpP*	LSC	71	665	292	801	252
*petB*	LSC	6	754	642		
*petD*	LSC	8	734	481		
*rpl16*	LSC	9	971	399		
*rpl2*	IRb	390	664	432		
*ndhB*	IRb	777	699	756		
*rps12*	IRb	229	—	26	544	126
*trnI-GAU*	IRb	37	941	35		
*trnA-UGC*	IRb	38	815	35		
*ndhA*	SSC	558	1,122	540		
*trnA-UGC*	IRa	38	815	35		
*trnI-GAU*	IRa	37	941	35		
*ndhB*	IRa	777	699	756		
*rpl2*	IRa	390	664	432		
*trnA-UGC*	IRa	38	815	35		
*trnI-GAU*	IRa	37	941	35		

### 3.2 Repetitive sequence variations analysis

For the sequence analysis, 40 repeats were detected in the cp genome in total, comprising 21 forward (F), 1 reverse repeat (R), 18 palindromic (P) repeats. No complement (C) repeat was marked out (Table 3). The fragment size of the repeat sequence was between 30 and 26,559 bp, of which 39 fragments were between 30 and 63 bp, and only 1 was the longest palindrome repeat (26,559 bp). Meanwhile, the longest forward repeat was 63 bp, positioned in the *accD* gene of the LSC region. Whereas the shortest repeat was 30 bp, mainly found in the *ycf2* gene of LSC and IRs regions, containing 5 palindromic repeats and 7 forward repeats. Meanwhile, we found that 19, 15, and 5 repeats were distributed within the LSC, IR, and SSC regions, respectively. Moreover, the following genes possessed the most repeats: *accD*, *ycf1*, *ycf2*, *ycf3*, *trnS-GCU*, *trnS-UGA*, *trnG-UCC*, *psaA,* and *psaB*. Furthermore, *ycf2* had the largest number of seven repeats, situated in the IR region. Therefore, we could find that the *ycf2* gene sequence difference in the IR region was more abundant than the difference in SSC and LSC regions.

**Table 3:** Distribution of repeat sequences in cp genes of *A. cochinchinensis*

ID	Repeat I start	Repeat II start	Type	Size (bp)	Distance	*E*-value	Gene	Region
1	59,144	59,174	F	63	–3	8.75 × 10^−23^	*accD*; *accD*	LSC; LSC
2	29,804	29,804	P	56	0	1.34 × 10^−24^	IGS	LSC; LSC
3	59,192	59,228	F	54	–3	1.43 × 10^−17^	*accD; accD*	LSC; LSC
4	129,395	129,410	F	54	–3	1.43 × 10^−17^	*ycf1; ycf1*	SSC; SSC
5	59,157	59,187	F	50	–2	6.04 × 10^−17^	*accD; accD*	LSC; LSC
6	90,608	90,629	F	49	–1	3.22 × 10^−18^	*ycf2; ycf2*	IRb; IRb
7	90,608	151,725	P	49	–1	3.22 × 10^−18^	*ycf2; ycf2*	IRb; IRa
8	90,629	151,746	P	49	–1	3.22 × 10^−18^	*ycf2; ycf2*	IRb; IRa
9	151,725	151,746	F	49	–1	3.22 × 10^−18^	*ycf2; ycf2*	IRa; IRa
10	59,162	59,228	F	48	–3	4.09 × 10^−14^	*accD; accD*	LSC; LSC
11	39,152	41,376	F	47	–3	1.53 × 10^−13^	*psaB; psaA*	LSC; LSC
12	59,140	59,236	F	40	–2	4.03 × 10^−11^	*accD; accD*	LSC; LSC
13	44,079	100,383	F	39	–2	1.53 × 10^−10^	*ycf3; IGS*	LSC; IRb
14	44,079	141,981	P	39	–2	1.53 × 10^−10^	*ycf3; IGS*	LSC; IRa
15	125,635	125,635	P	39	–3	5.67 × 10^−9^	IGS	SSC; SSC
16	127,026	127,026	P	39	–3	5.67 × 10^−9^	*ycf1; ycf1*	SSC; SSC
17	129,395	129,425	F	39	–3	5.67 × 10^−9^	*ycf1; ycf1*	SSC; SSC
18	8,775	8,775	P	38	0	9.19 × 10^−14^	IGS	LSC; LSC
19	59,140	59,200	F	37	–2	2.20 × 10^−9^	*accD; accD*	LSC; LSC
20	32,182	32,201	P	35	–3	1.04 × 10^−6^	IGS	LSC; LSC
21	8,228	45,494	P	33	–2	4.47 × 10^−7^	*trnS-GCU; trnS-GGA*	LSC; LSC
22	115,811	115,811	R	33	–2	4.47 × 10^−7^	IGS	SSC; SSC
23	69,831	69,847	F	32	–2	1.68 × 10^−6^	IGS	LSC; LSC
24	8,226	35,982	F	32	–3	5.04 × 10^−5^	*trnS-GCU; trnS-UGA*	LSC; LSC
25	35,985	45,494	P	32	–3	5.04 × 10^−5^	*trnS-UGA; trnS-GGA*	LSC; LSC
26	9,961	36,985	F	31	–3	1.83 × 10^−4^	*trnG-UCC; trnG-UCC*	LSC; LSC
27	90,630	90,651	F	30	–2	2.36 × 10^−5^	*ycf2; ycf2*	IRb; IRb
28	90,630	151,722	P	30	–2	2.36 × 10^−5^	*ycf2; ycf2*	IRb; IRa
29	90,651	151,743	P	30	–2	2.36 × 10^−5^	*ycf2; ycf2*	IRb; IRa
30	44,091	100,395	F	30	–3	6.60 × 10^−4^	*ycf3; IGS*	LSC; IRb
31	44,091	141,978	P	30	–3	6.60 × 10^−4^	*ycf3; IGS*	LSC; IRa
32	87,962	87,985	F	30	–3	6.60 × 10^−4^	IGS	IRb; IRb
33	87,962	154,388	P	30	–3	6.60 × 10^−4^	IGS	IRb; IRa
34	87,985	154,411	P	30	–3	6.60 × 10^−4^	IGS	IRb; IRa
35	90,606	90,648	F	30	–3	6.60 × 10^−4^	*ycf2; ycf2*	IRb; IRb
36	906,06	151,725	P	30	–3	6.60 × 10^−4^	*ycf2; ycf2*	IRb; IRa
37	90,648	151,767	P	30	–3	6.60 × 10^−4^	*ycf2; ycf2*	IRb; IRa
38	151,722	151,764	F	30	–3	6.60 × 10^−4^	*ycf2; ycf2*	IRa; IRa
39	154,388	154,411	F	30	–3	6.60 × 10^−4^	IGS	IRa; IRa

Note: F = forward, P = palindromic, R = reverse, C = complement, IGS = the intergenic spacers; Minimum length: 30 bp; Hamming distance: 3; Soft: vmatch-2.3.0.

**Figure 2:**
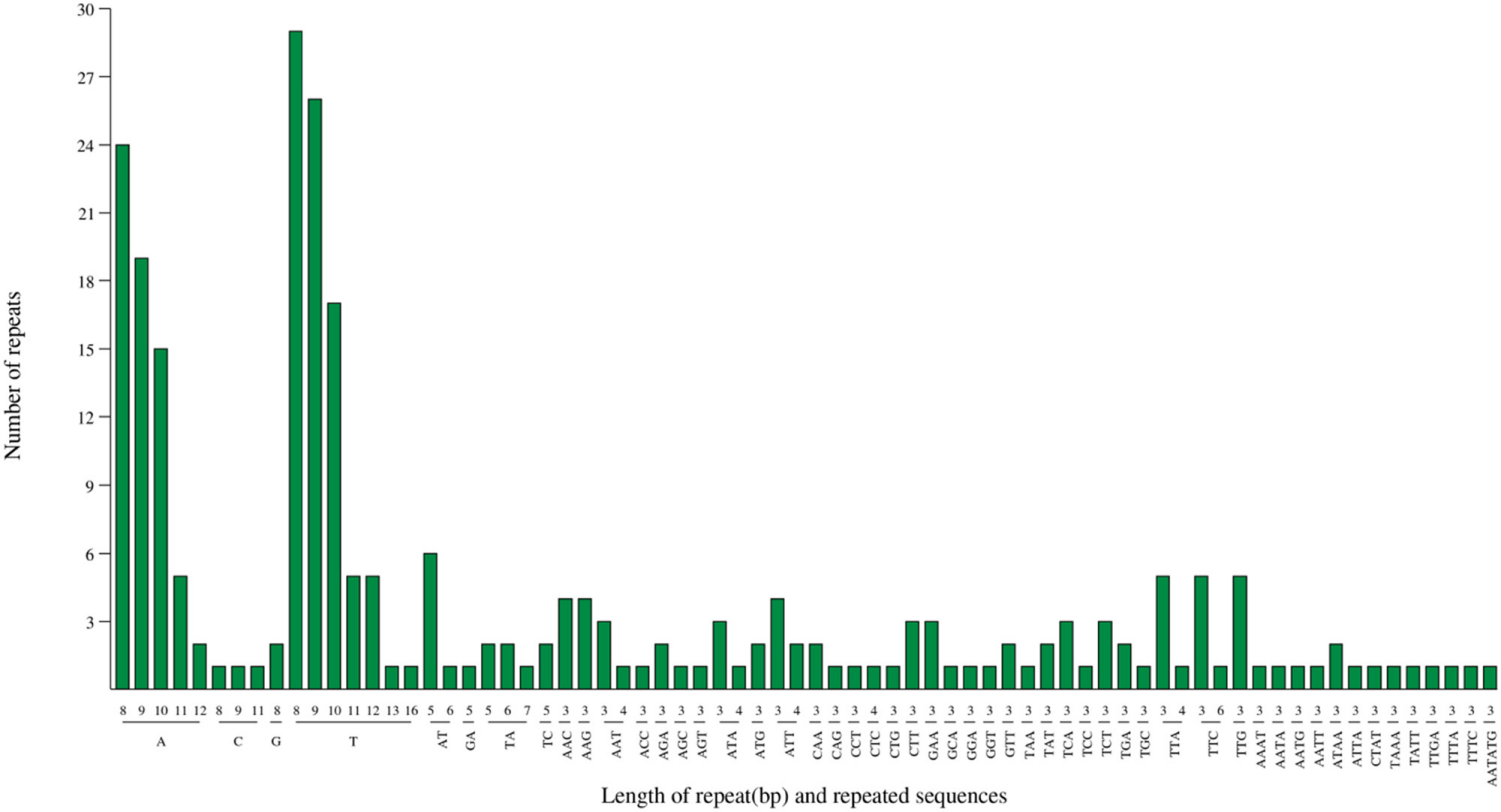
The type and number of SSR motif in *A. cochinchinensis* cp genome.

In addition, SSR repeats were analyzed systematically in *A. cochinchinensis* cp genome. Six categories of SSRs (1–6 bp repeat) were noted, and a total of 247 SSR loci were detected, most of which were scattered in LSC (65.20%) and SSC (17.0%) regions. The SSR numbers were 161, 42, and 44, located in LSC, SSC, and IR regions individually. For the SSR repeat types, 154 mono-nucleotide repeats were dominated (59.69%), followed by 76 trinucleotide repeats, 15 dinucleotide repeats, 13 tetranucleotide, 1 hexanucleotide, and without pentanucleotide repeats (Figure 2). An inverse relationship was verified between the length and the abundance of repeat sequence units.

In addition, 122 SSRs were detected in gene internal structure, including *psbA*, *matK*, *trnK-UUU*, *rps16*, *trnG-UCC*, *atpF*, *atpI*, *rpoC2*, *rpoC1*, *rpoB*, *psbC*, *psaB*, *psaA*, *ycf3*, *ndhK*, *trnV-UAC*, *accD*, *ycf4*, *cemA*, *petA*, *psaJ*, *rps18*, *clpP*, *psbB*, *petB*, *rpl16*, *rpl22*, *rps19*, *ndhB*, *rrn16*, *trnI-GAU*, *rrn23*, *ndhF*, *ccsA*, *ndhD*, *ndhE*, *ndhA*, *ycf1*, *rrn23*, *trnI-GAU*, *rrn16*, *ndhB*, *ycf2*, *rpl2*, and *rps19*. Furthermore, the *ycf2* gene had the largest number of 18 SSRs. The remaining 125 SSRs were detected in intergenic regions. For the repeat motifs, there were 65 A (25.09%), and 84 T (32.43%) repeats in the single nucleotide repeats; in the dinucleotide type, 12 AT/AT (4.63%) repeats were marked out; and the trinucleotide repeat contained 23 AAT/ATT (8.88%) and 21 AAG/CTT (8.11%). The results suggested that the use of SSR in *A. cochinchinensis* cp genome was characterized by high A/T frequency, consistent with the high AT content in the genome.

### 3.3 CUB analysis

**Figure 3:**
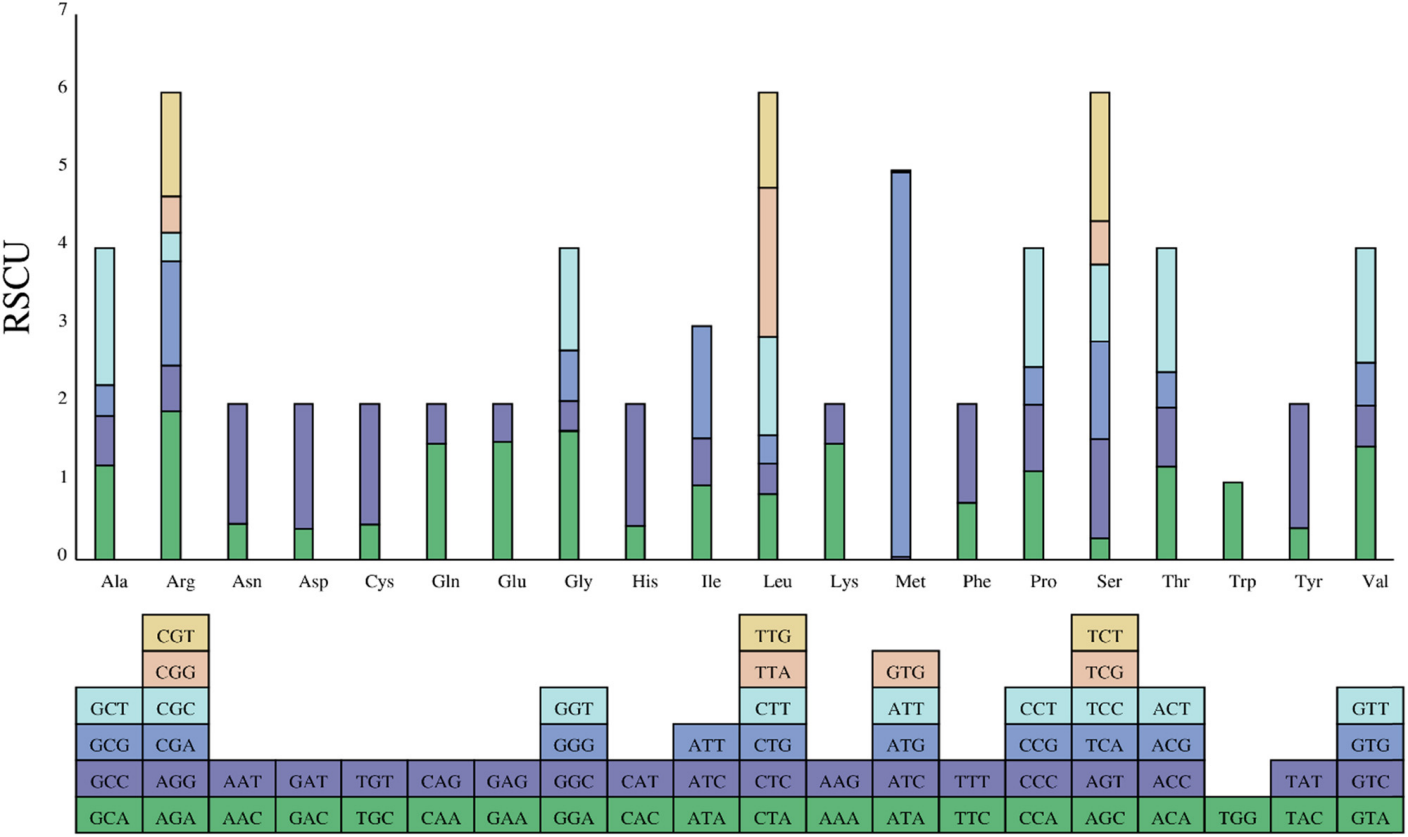
The histogram of RSCU value in *A. cochinchinensis* cp genome. Note: The *x*-axis represents amino acids, the box below represents all codons corresponding to each amino acid, and the height of the *y* axis represents the sum of RSCU values for all codons.

There were 76,812 nucleotides and 25,604 codons in all protein-coding genes. Leucine (2,643 codons, accounting for 10.63%) was the most abundant amino acid. Isoleucine was the second most abundant amino acid, which accounted for 8.69% (2,217 codons). However, cysteine was the least abundant amino acid with only 1.16% (298 codons) (Figure 3). The recognition pattern of the codon-anticodon showed that all amino acids were associated with 28 tRNAs containing codons. The initiation codon AUG had the RSCU value of 4.935. The RSCU value for the termination codons (UAG, UGA, and UAA) were 0.8721, 0.6978, and 1.4301, respectively. Thirty-one codons had RSCU value greater than 1, among which 28 ended with A or U and 3 ended with G or C. The result showed that the codon usage of A or U (T) was preferred to those of G or C.

### 3.4 IR boundary analysis

**Figure 4:**
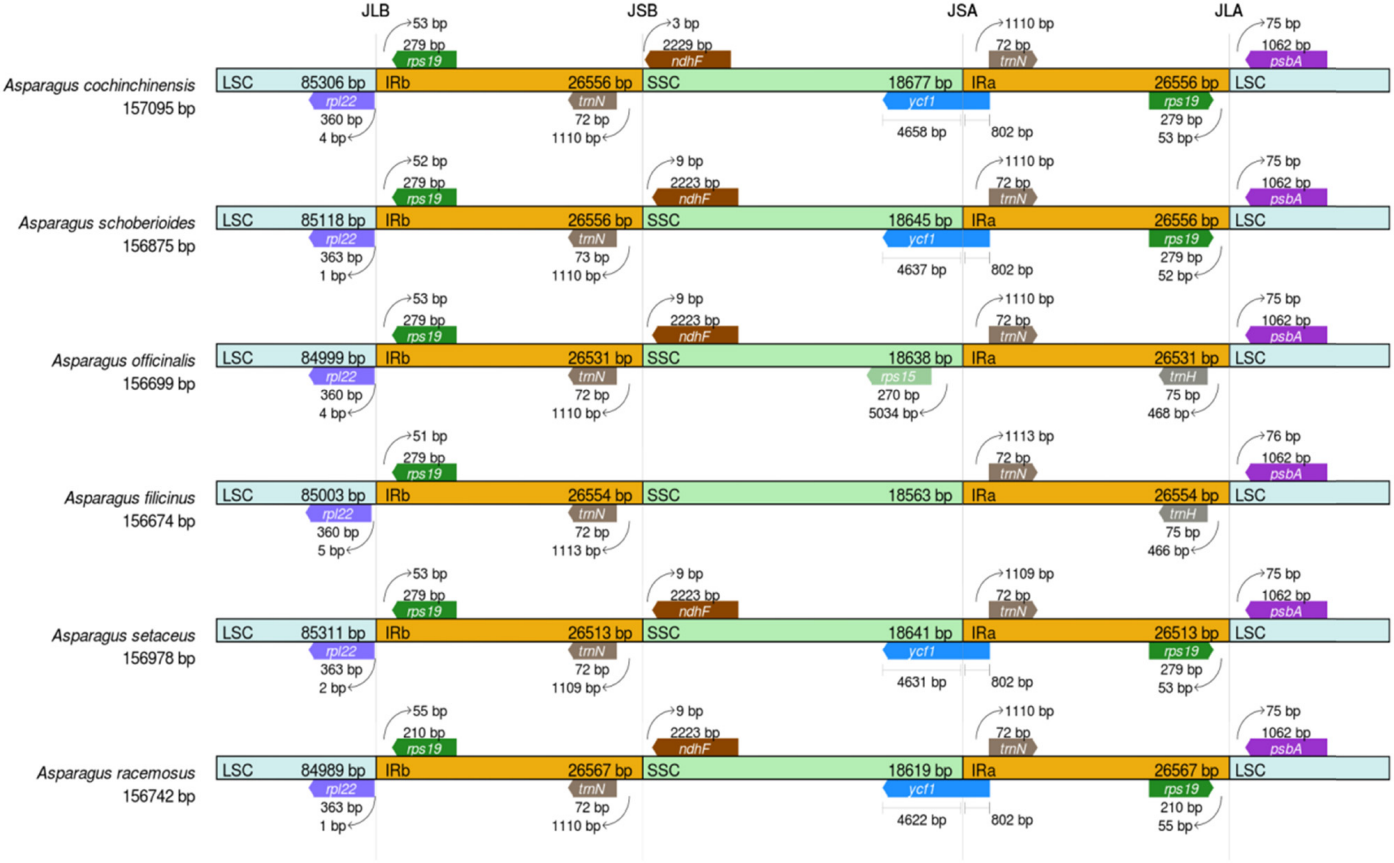
Boundary difference analysis of the quadripartite structure containing LSC, IRb, SSC, and IRa region of *A. cochinchinensis* and other five cp genomes of the genus *Asparagus*.

The boundaries were compared, including IR, LSC, and SSC regions of the cp genome in six *Asparagus* species, and the cp genome of *A. officinalis* L. was set as a reference. It was shown that the LSC-IRb joining regions of six species were similar, and they all contained *rpl22* and *rps19* genes. There were differences in IRb-SSC joining region. No gene existed on the right boundary of *A. filicinus*. In the SSC-IRa joining region, the left gene of *A. officinalis* was *rps15*, there was no gene on the left of *A. filicinus*, and the left gene was *ycf1* in other species. In the IRa-LSC joining region, the gene was *trnH* gene on the left side of *A. officinalis* and *A. filicinus*, and the gene exhibited in other species was *rps19* (Figure 4). It can be seen that the interface positions and gene types of LSC, SSC, IRa, and IRb joining areas were almost the same in the genome of the genus *Asparagus*, which had the characteristics of conservation.

### 3.5 Collinearity analysis of cp genomes in *Asparagus* species

**Figure 5:**
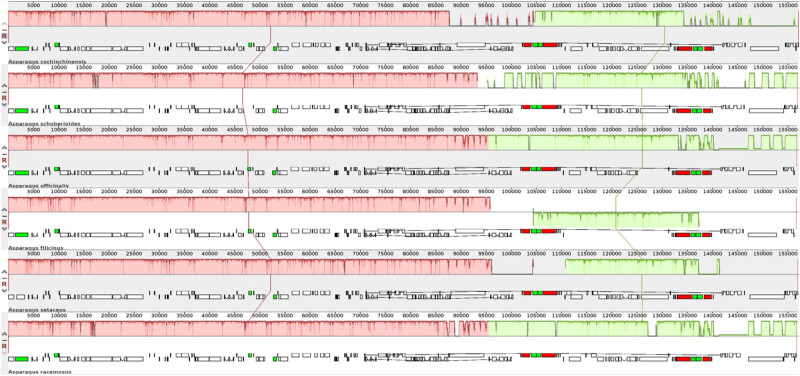
Collinearity analysis of cp genomes among six *Asparagus* species using Mauve (http://darlinglab.org/mauve). **Note:** The collinear regions are shown in the same color as rectangular blocks. The rectangles indicate similarities among genomes, and the lines represent a collinear relationship between rectangles. The short squares represent the gene position of each genome. Genome regions are colored as CDs (white), tRNA (green), and rRNA (red).

To detect the gene sequence divergence and provide an illuminating insight into the evolutionary mechanism of the genus *Asparagus*, we aligned the complete genomes of six *Asparagus* species with the Mauve program [14]. The length of the six analyzed cp genomes varied from 156,674 bp (*A. filicinus*) to 157,095 bp (*A. cochinchinensis*). It was shown that a similar gene order was shared between *A. cochinchinensis* cp genome and other *Asparagus* species (Figure 5). Therefore, we can speculate that a highly conservative cp genome content, genetic structure, and gene order were exhibited in the genus *Asparagus*. And no translocations or inversions were shown in the *Asparagus* species. Therefore, it was shown that the LSC and SSC regions were less conserved than IR regions.

### 3.6 Ka/Ks substitution rate analysis

**Figure 6:**
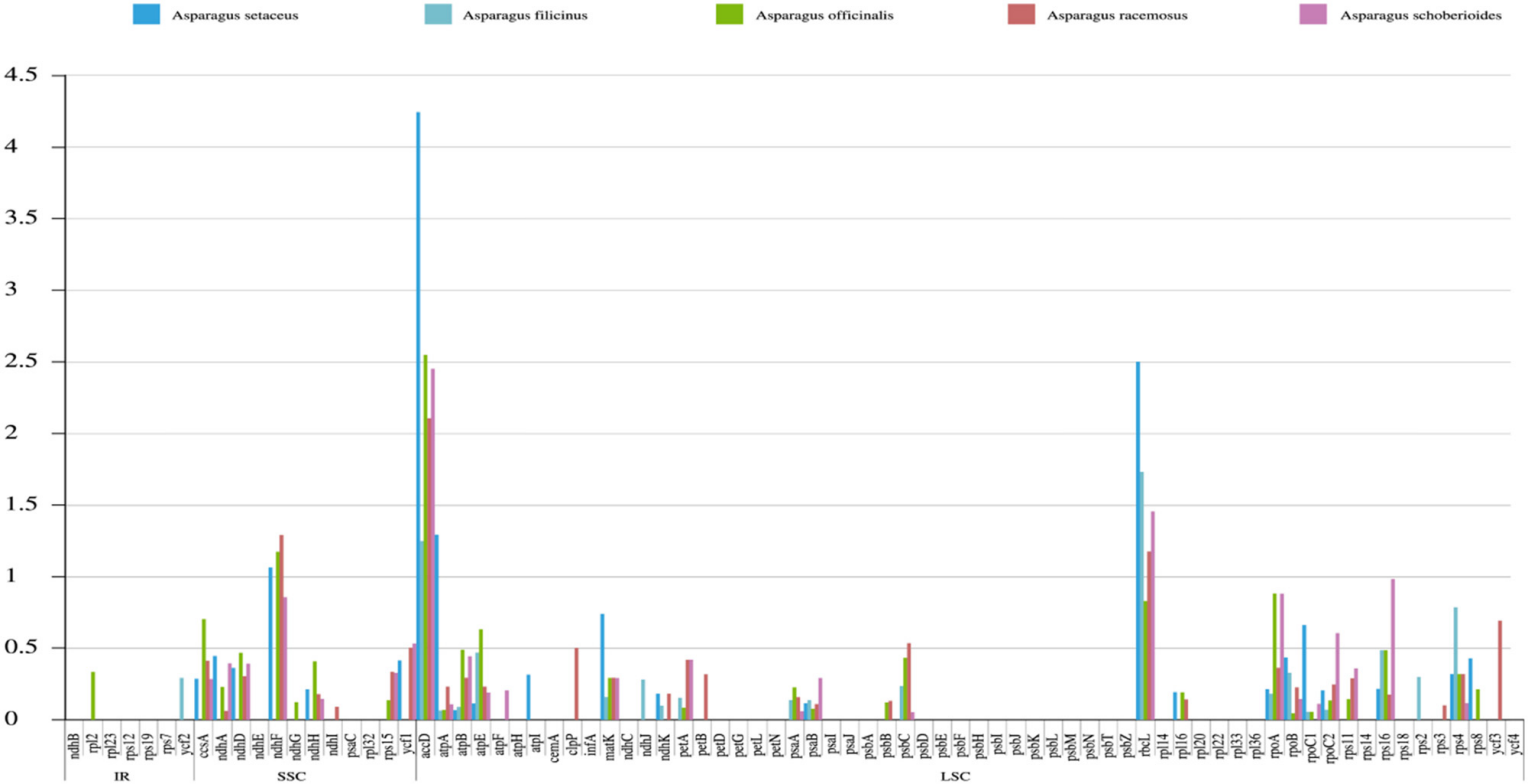
The Ka/Ks ratio of 84 protein-coding genes among the *Asparagus* cp genomes, including *A. cochinchinensis* and five other reported species.

In order to reveal the gene characteristics, the Ka/Ks substitution ratio of protein-coding genes was calculated among the six *Asparagus* species. In total, 84 identical genes could be used for the diversity analysis. The 57.14% of protein-coding genes (48 of 84 genes) had an average Ka/Ks ratio ranging from 0 to 4.23873 (Figure 6). The Ks value (0.0277079) and Ka/Ks ratio (4.236) of *accD* were both the highest, implying that *accD* evolved at a fast-rate. Meanwhile, 4 hypervariable regions were identified including (*rbcL*, *accD*, *ndhF*, and *atpA*), and their Ka/Ks value were all greater than 1. The results showed that only 4 genes were positively selected and the other 80 genes were negatively selected or neutrally changed.

### 3.7 The nucleic acid diversity (PI) value in *A. cochinchinensis* cp genome

**Figure 7:**
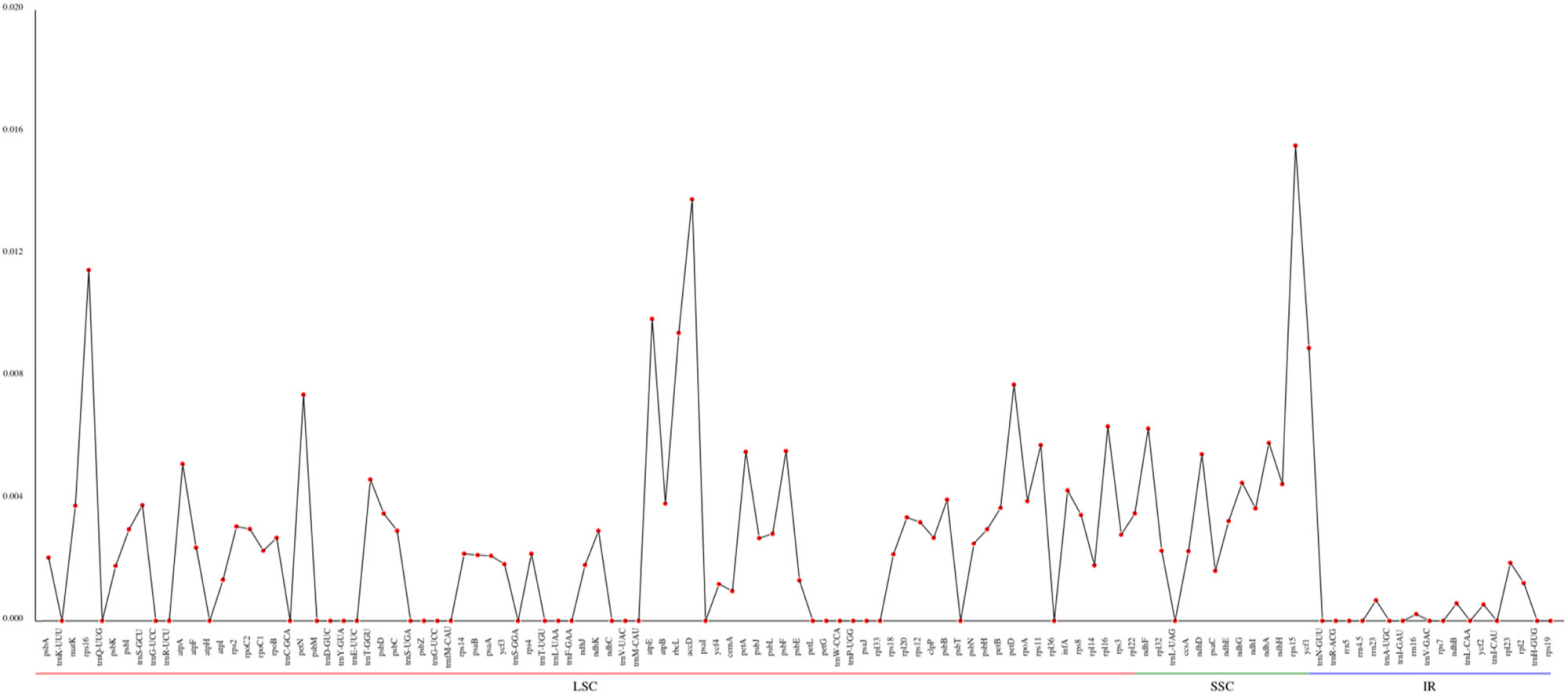
The PI value of the *A. cochinchinensis* cp gene. Note: The gene name is shown in abscissa, and the PI value is shown in ordinate.

To detect the divergence hotspot, polymorphism index (PI) value was calculated by DnaSP v5.10 with the sliding window method [19]. In total, 351 single nucleotide polymorphisms and 217 insertion and deletions were marked out in the six *Asparagus* species. The average value of PI was 0.002394, and three highly variable heterotopic sites with high PI values (PI > 0.01) were accurately located (*rps16*, *accD*, and *rps15*) (Figure 7). In addition, 35 polymorphism areas had more than 5 mutations, among which *ycf1* had the largest mutation site number of 93. Furthermore, the areas which were rich in mutation sites include *rps16*, *accD*, *psaI*, *matK*, *rps15*, *rps12*, *ndhF*, *rpl32,* and *ycf1*. Thus, the above-identified highly variation sites can provide useful molecular information for species identification, evolutionary relationship, and genetic diversity analysis in the genus *Asparagus*.

### 3.8 Phylogenetic analysis of *A. cochinchinensis* cp genome

**Figure 8:**
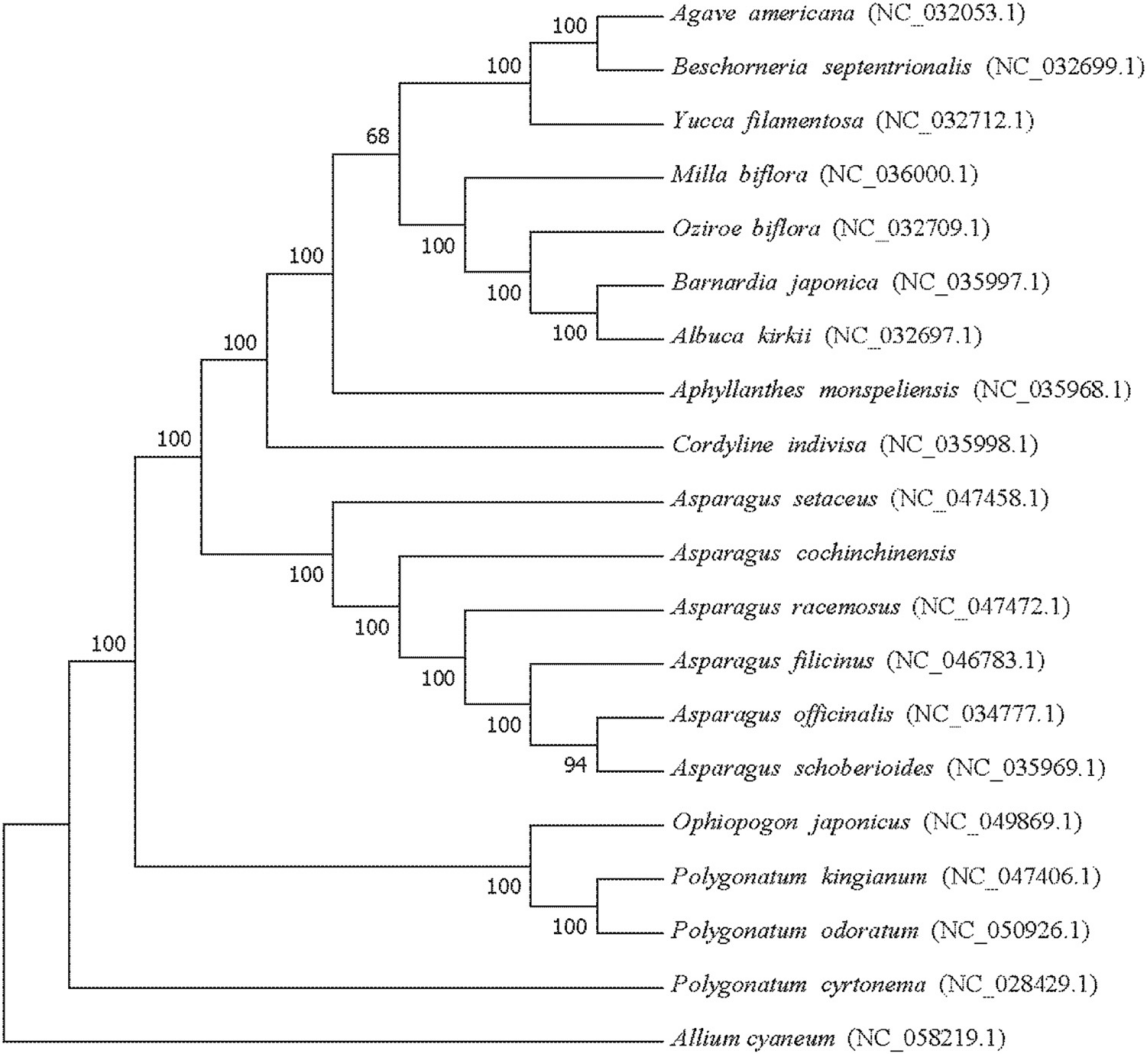
The phylogenetic tree was reconstructed with the entire cp genomes by the ML method, and *Allium cyaneum* (NC_058219.1) was used as the outgroup.

According to the phylogenetic tree analysis of 19 complete cp genome sequences in Asparagaceae (Figure 8), we can further clarify the taxonomic status and phylogenetic development relationship of *A. cochinchinensis*. The phylogenetic tree could clearly divide the analyzed species into five branches. The first major branch referred to the three species (*Agave Americana*, *Beschorneria septentrionalis*, and *Yucca filamentosa*) of the subfamily Agavoideae, the second major branch included the subfamily Brodiaeoideae (*Milla biflora*) and the subfamily Hyacinthaceae (*Oziroe biflora*, *Barnardia japonica*, and *Albuca kirkii*), and the third major group contained the subfamily Aphyllanthoideae (*Aphyllanthes monspeliensis*) and the subfamily Aphyllanthoideae (*Cordyline indivisa*). The fourth branch had six species of *Asparagus* in Asparagoideae including *A. setaceus, A. cochinchinensis, A. racemosus, A. filicinus, A. officinalis,* and *A. schoberioides*, and the fifth branch owned four species (*Ophiopogon japonicas*, *Polygonatum kingianum*, *Polygonatum odoratum*, and *Polygonatum cyrtonema*) of the subfamily Pseudophyllaceae. The results showed that *A. cochinchinensis* was closer to *Asparagus racemosus* in the genus *Asparagus*. Therefore, the elucidation of molecular evolutionary relationship in *A. cochinchinensis* was more conducive to the study of phylogenetic relationships and species identification within the genus.

## 4 Discussion

### 4.1 Genome organization

In this research, the whole cp genome of *A. cochinchinensis* was sequenced with Illumina high-throughput platform. The results from the cp genome analysis showed that the complete plant cp genome sequence could be obtained by direct extraction of whole genome DNA, homology comparison with Blast analysis of similar sequences in related species, and DNA sequence assembly with cp splicing software, which set a good reference for the study of other species. The analysis of cp structure characteristics indicated that *A. cochinchinensis*, like other *Asparagus* plants, had a typical circular structure consisting of one SSC region, one LSC region, and a pair of inverted repeats (IRa and IRb). This was similar to the cp genome structure of most angiosperms, further demonstrating the conservation of cp genome structure [20,21].

By comparing the *Asparagus* cp genomes, including *A. filicinus*, *A. officinalis*, *A. racemosus*, *A. schoberioides,* and *A. setaceus*, the genome size of the genus *Asparagus* was found to be 156,674 (*A. filicinus*) to 157,095 bp (*A. cochinchinensis*). The cp genome size of terrestrial plants was usually around 120–160 kb [22]. The gene number of around 110–140 in angiosperms cp genome was relatively stable [23]. And the total gene number annotated in cp genome of *A. cochinchinensis* was 137, which consisted of 86 protein coding genes, 8 rRNAs, and 38 tRNAs. At the same time, its gene number, types, and structure of genes in its cp genome were very similar with *A. setaceus*, indicating that the genus *Asparagus* evolution was relatively slow, and its GC content had the characteristics of a typical angiosperm cp genome [24].

### 4.2 SSR and repeat sequence

The repetitive sequences are normally considered to be homologous DNA fragments that are found in many copies present in the genome. The DNA repeat sequences make up 90% of the genome size in higher plants, which are helpful for the evolutionary analysis of plant phylogeny [25]. The cp genome is maternal inheritance, which is conservative and simple in its structure. And the SSR is an efficient molecular marker, which is widely used in genetic breeding and population genetics [26]. In total, 247 SSRs and 5 classes of repeats were identified by SSR analysis in *A. cochinchinensis* cp genome. It was found that a higher content of A or T (67.52%) was exhibited in all nucleotide repeats, giving rise to the base composition bias [27]. This phenomenon was in accordance with the adenine-thymine (A-T) richness (62.52%) of the cp genome and agreed with the observation that the cp SSRs often consisted of poly-A and poly-T types [28]. It was speculated that A-T conversion was easier than G-C in the cp genome.

Repeat sequence affects gene transcription regulation, protein translation, and chromosome formation and profoundly impacts the evolution, inheritance, and variation in genes in different species. Repeat sequence diversity is the main reason for fragment duplication, deletion, and rearrangement in the cp genome [29]. The repetitive sequences of *A. cochinchinensis* were mainly forward and palindromic repeats, and there was no complementary repeat sequence. It was also reported that there were no complementary and reverse repeats in the cp genome of *Sonchus brachyotus* [30]. Differences in repeat sequence showed that the dissimilarity in the type and number could reflect the difference between the mutation frequency and evolution rate of inter-species.

### 4.3 Codon analysis

Codon usage is a key factor influencing the cp genome evolution and the genetic information expression [31]. The RSCU value was computed in the cp genome of *A. cochinchinensis*. There were 31 codons with an RSCU value greater than 1.00, of which 28 ended with A or U, indicating that the cp genome codons of *A. cochinchinensis* preferred to end with A or U (T) and did not prefer to end with G or C. The number of codons encoding leucine (Leu) was the largest, with 2,643. The results showed that the cp genome of *A. cochinchinensis* was similar to most angiosperms and tended to use AT-terminal codons.

### 4.4 Structure variation analysis

The change in IR/SC boundary position among different species is a common phenomenon in the cp genome, and the boundary position changes are usually different in these species of the same family [32]. The result showed that the reported cp genomes of six species in the genus *Asparagus* were relatively conservative in its structure and size, the boundary positions of IR and SSC had fewer variations among different species. Compared with the other five species, the IR area (26,513 bp) of *Asparagus setaceus* was the smallest among the six species, which was mainly manifested in the *rpl2* gene of LSC/IR area. In *A. cochinchinensis*, the length of 718 bp in *rpl2* gene extended to LSC region, while the *rpl2* gene in other *Asparagus* species was completely located in IR region. Therefore, the change in LSC/IRb boundary in the genus *Asparagus* was the main reason for the contraction and expansion of IRs region.

The Ka/Ks value analysis is an effective method to evaluate whether protein-coding genes have adaptive evolution [33]. The synonymous nucleotide substitutions of most genes in organisms occur more frequently than the non-synonymous substitutions, so the Ka/Ks value is usually less than 1 [34]. In this study, four positive selection genes (*rbc*L, *accD*, *ndhF*, and *atpA*) were detected, and their Ka/Ks values were greater than 1, indicating that the four genes were undergoing rapid evolution in recent years. In-depth study of the above genes had certain significance for the evolution of the genus. Through the sequence comparison of the whole genome, it was found that *rps1*6, *accD*, *psaI*, *matK*, *rps15*, *rps12*, *ndhF*, *rpl32*, and *ycf1* had abundant variation sites, among which there were variations that caused gene shift mutation such as *ycf1*. This suggested that the gene may not function conservatively in evolution, perhaps its function had changed or been lost [35]. Therefore, molecular markers could be developed for the identification of different germplasm resources in the genus *Asparagus* by comparing the regions with large variations (such as *accD*, *rps15*, *rps12*, *ndhF*, and *ycf1*).

### 4.5 Phylogenetic relationship analysis

The phylogenetic tree of 19 species in 7 subfamilies was constructed using the cp genome sequence of Asparagaceae plants. The results showed that the phylogenetic tree had a clear classification relationship, and the bootstrap values were above 94. Each node had a high value, and each subfamily showed a clear evolutionary relationship. The studies of Sheng et al. [36] and Raman et al. [37] also supported the systematic classification results of the section in the family. However, in the systematic classification of the genus *Asparagus* species, the genetic relationship between *A. cochinchinensis* and *A. racemosu*s was the closest, which was inconsistent with the genetic relationship between *A. cochinchinensis* and *A. officinalis* obtained by Norup et al. [38]. The cp genome sequence was used in this study, while the three plastid regions (*trnH-psbA*, *trnD-T*, and *ndhF*) and the phytochrome C gene were used in Norup’s study, which may be different from the data types used. Studies have shown that phylogenetic analysis can obtain more accurate evolutionary relationships by using the entire genome sequence [39,40]. Therefore, in order to explore the phylogenetic relationship of the genus *Asparagus*, it is necessary to sequence more related species and obtain more sequence information to carry out phylogenetic research in future.

## 5 Conclusion

In this study, based on the sequencing method of plant genome DNA using high-throughput sequencing technology, the similarity comparison with related species, and the assembly of cp splicing software, the complete cp genome of *A. cochinchinensis* was successfully assembled and annotated, which provided a reference for the cp genome research of other species. The genome structure, gene sequence, GC content, and codon preference of *A. cochinchinensis* cp genome were similar to five reported species of Asparagus gen*us*. In its cp genome, abundant genome repeats were identified, and polymorphic variation sites were detected. The phylogenetic analysis of cp genome confirmed the phylogenetic position of A. cochinchinensis. The sequencing and analysis of its cp genome provide data basis for phylogenetic analysis of the genus *Asparagus*. As a traditional medicinal plant, *A. cochinchinensis* has important medicinal and economic value. In order to identify the authenticity of the traditional Chinese medicine species, we can utilize the obtained repeat sequence and polymorphism sites to be devised as molecular barcodes in the next research.
